# Protective reactions of ICU nurses providing care for patients with COVID-19: a qualitative study

**DOI:** 10.1186/s12912-021-00567-6

**Published:** 2021-03-17

**Authors:** Yaser Moradi, Rahim Baghaei, keyvan Hosseingholipour, Farzin Mollazadeh

**Affiliations:** Patient Safety Research Center, Clinical Research Institute, Nursing and Midwifery School, Urmia University of Medical Sciences, Urmia, Iran

**Keywords:** Nurse, Protective reactions, COVID-19, Qualitative study

## Abstract

**Background:**

The exponential spread of COVID-19 has caused a huge threat to public health worldwide. Providing care for patients with COVID-19 is a stressful experience for ICU nurses, which can affect their protective reactions. The present study was conducted to explore the protective reactions of ICU nurses providing care for patients with COVID-19**.**

**Methods:**

This qualitative descriptive study was conducted to discover the protective reactions of nurses providing care for patients with COVID-19. A total of 14 ICU nurses were selected by purposive sampling. Data were collected using individual semi-structured face-to-face interviews. All interviews were recorded, and then codes and themes were extracted using content analysis method.

**Finding:**

Seventeen subcategories, six categories and two themes were extracted from the analysis of data. These themes include “Unbalanced self-protective reactions” and “Responsible self-protective reactions”.

**Conclusion:**

During the COVID-19 epidemic and crisis, ICU nurses exhibit different self-protective reactions when providing care for patients with COVID-19, which include unbalanced and responsible reactions. Nursing managers can mitigate these unbalanced reactions by identifying them and their roots. Identifying the protective reactions of ICU nurses in providing care for patients with COVID-19 could assist in developing the necessary interventions to promote positive reactions and reduce unbalanced reactions by finding their root causes.

**Supplementary Information:**

The online version contains supplementary material available at 10.1186/s12912-021-00567-6.

## Background

In late December 2019, a new Coronavirus called the Novel Coronavirus-2019 (2019-nCoV) initiated the spread of pneumonia from Wuhan (Hanan’s seafood market) throughout China, which has now caused a huge threat to public health worldwide [1]. With symptoms such as cough, fever, and in more severe cases, pneumonia, this virus causes respiratory illness [2]. The Coronavirus that causes COVID-19 is referred to by the International Committee on Virus Classification the Acute Respiratory Syndrome of Coronavirus-2 (2-SARS-CoV); an enveloped virus belonging to the Coronavirus-β, often polymorphic, in round or oval shaped particles of 60-140 nm in diameter. This virus has a large bulb-like margin resembling images of a royal crown or solar corona [3].

On March 11, 2020, the World Health Organization announced that the spread of COVID-19 has reached a global pandemic level [2]. According to the official statistics published in the Worldometer website until May 10th, confirmed cases are 4,121,818 in the world and 106,220 in Iran, of whom, 280,868 people in the world and 6589 people in Iran have died, and 1,451,444 people in the world and 85,064 people in Iran have recovered. The global mortality rate of 16% has been registered for this disease, and 2% of all patients with COVID-19 worldwide are in a critical condition and need special cares [4].

No specific treatment has yet been found for this disease, and treatments are supportive, and rather emphasize the prevention and reduction of transmission [5]. because this virus is highly communicable, and appears to be transmitted through breathing particles spread by coughs and sneezes, just like influenza and rhinovirus. Moreover, aerosol transmission can also occur with prolonged exposure in closed environments containing high aerosol density. Hence, healthcare providers are at a greater risk than others [5, 6].

According to the World Health Organization approach, observing breathing and hand hygiene, and using Personal Protective Equipment (PPE) are appropriate for healthcare providers [6]. The National Center for Infectious Diseases has recommended that healthcare providers use gloves, gown, respiratory protections (disposable N95 respirators), goggles (disposable safety goggles and face protectors) when faced with a patient with known infection or suspected of COVID-19 [7, 8]. Meanwhile, nurses and especially ICU nurses are at the frontline of the fight against Coronavirus and amid the crisis; the increasing number of hospitalized patients, shortage of nursing workforce, shortage of PPE, fear of being infected and transmitting the infection to family members concerns them and can affect how they care for patients [9]. Furthermore, the exponential spread of this virus throughout the world and the increasing need for PPE has turned the lack of PPE into a major global challenge. Healthcare providers do not have sufficient access PPE because those who are not in direct contact with the patients use them, therefore, lack of access and improper use of PPE have created challenges for healthcare providers, especially nurses, which can disrupt the provision of safe patient care and occasionally even death of these patients [10–13].

The stress of providing cares for a COVID-19 patient plus the lack of access to appropriate PPE, as well as limited provision of PPE can lead to particular reactions from ICU nurses to protect themselves against COVID-19. Exploring these protective reactions based on their lived experiences can help understand the strategy used by them in such a crisis, and also enables healthcare authorities to understand this phenomenon and take necessary measures to improve the quality and quantity of the nurses’ PPE. The present qualitative descriptive study was conducted to explore the protective reactions of ICU nurses when providing care for patients with COVID-19.

## Methods

### Study design

In the present study, qualitative descriptive approach was used to understand the protective reactions of ICU nurses providing care for patients with COVID-19. Some qualitative studies do not have a specific methodological or discipline. Such studies provide a comprehensive short description of an event [14].

### Participants and setting

A total of 14 ICU nurses were selected by purposive sampling. Maximum diversity was considered in the selection of participants in terms of personal details (age, gender, education, and work history) to obtain a wide range of experiences.

The study inclusion criteria were: ICU nurses with at least 1 year’s experience in ICU, experience of providing care for patients with COVID-19, no history of infection with COVID-19, willingness to take part and share their experiences.

### Data collection

Data were collected through individual semi-structured face-to-face interviews (Additional file 1). The participants were asked to share their experiences in relation to protective reactions in dealing with patients with COVID-19. Further questions were asked based on descriptions provided by the participants. Examples of the interview questions are as follows:

“*Describe your protective behaviors when providing care for a COVID-19 patient?” Give an example*.

“Does the way you provide care change when dealing with patients with COVID-19?” please explain.

The researcher conducted all interviews by complying with personal protection principles and coordination with the participants in classes available in clinical fields, and tried to maintain the privacy and the greatest comfort for the participants. With their consent, the researcher recorded the participants’ narratives during interviews using an audio recorder. Each interview lasted 40 min. Interviews continued until saturation of data.

### Data analysis

Data were analyzed using content analysis based on Granheim & Lundman method [15]. After each session, the recorded contents of interviews were repeatedly and carefully listened to, transcribed on paper, and then typed in Microsoft Word®, and data were classified and analyzed in MAX. Q DA-10 R250412. The semantic units were identified and encoded after careful review of the transcribed materials. In this stage, the initial codes were generated as explicit and implicit codes. Then, codes were merged and categorized according to similarities. Attempt was made to make the most homogeneity within categories and the most heterogeneity between categories.

### Rigor

The researcher tried to establish a friendly relation with the participants and conduct interviews in a comfortable space. Furthermore, the researcher deeply engaged with the data, spent sufficient time to collect and analyze the data, and used member check and peer check.

### Finding

Of the 14 participating nurses, 10 were female and four were male, with a mean age of 33.5 years and work history of 8.21 years; three had Master’s degree, and the rest had Bachelor’s degree in nursing.

**Fig. 1:**
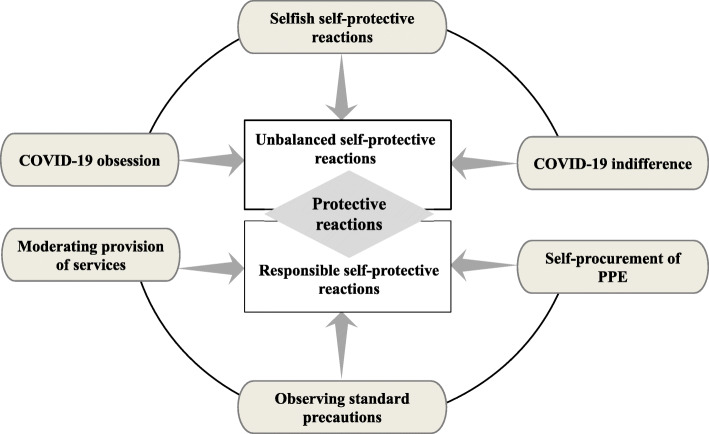
The protective reactions of ICU nurses providing care for patients with COVID-19

Analysis of data yielded 17 subcategories, six categories, and two themes (Table 1). These themes were “Unbalanced self-protective reactions” and “Responsible self-protective reactions”. The nurses’ protective reactions were defined as a self-protection reaction that could have been unbalanced (selfish, indifferent to COVID-19 and COVID-19 obsession), or responsible (moderation in providing care, self-provision of PPE, and observing standard precautions). Figure 1 depicts the general outline of Protective reactions of ICU nurses providing care for patients with COVID-19.

**Table 1:** Categories, subcategories, and codes extracted from data analysis

Unbalanced self-protective reactions	Selfish self-protective reactions	Only considering one’s own life
		Taking other people’s PPE
		Desire to avoid working with the patient
		Eagerness to be in non- COVID-19 wards
	COVID-19 obsession	Excessive fear of touching surfaces
		Suspecting everyone
		Excessive self-examination
		Obsessive behavior
	COVID-19 indifference	Entering a careless status
		Fed-up with wearing PPE
		Self-censoring
**Responsible self-protective reactions**	**Moderating provision of services**	Reducing duration of care
		Limiting contact with the patient
	**Self-procurement of PPE**	Purchasing PPE on the open market
		Demanding PPE from officials
	**Observing standard precautions**	Observing the principles of personal protection and safety
		Being careful in providing care

### Unbalanced self-protective reactions

The participants exhibited unbalanced self-protective reactions in providing care for patients with COVID-19 including selfish, indifferent to COVID-19 and COVID-19 obsessive self-protection. Regarding selfish self-protective experience, one of the nurses said:

“*Right now, everybody is thinking about their own life. Before COVID-19, when we had an extra workforce, the nursing office would ask the head nurse to send someone to another ward. Nobody wanted to go at all. But now, we are all eager to leave the ward and go to other wards.*” (Female nurse)

Another nurse commented:

“*There’s scarcely enough protective equipment. Colleagues quarrel with each other over protective equipment, without worrying which equipment is theirs. They take all available equipment for themselves and use them.*” (Male nurse).

The participants stated that providing care for a patient with COVID-19 has turned into a mental and behavioral obsession whether all protective principles have been observed or not. This obsession was described by many participants as an unpleasant experience. One of the nurses described her obsession with COVID-19 as follows:

“*We’ve become far too obsessive about everything. When the shift is over, it takes an hour for us to leave the ward; we disrobe and scrub. Our scrub is frightening. We think everything is infectious because of our obsession. Interestingly, it is the same at home, too.*” (Female nurse)

Another nurse explained:

“*We check ourselves excessively. Have I taken off the gown I was wearing properly? Has this gown touched another part of my body? Have I properly taken off my cap and gloves? One checks over oneself excessively. These have mentally exhausted us.*” (Male nurse).

Some participants had turned indifferent and careless due to an obsession-induced exhaustion. COVID-19 indifference refers to being fed up with wearing PPE and self-censorship. One of the nurses shared their experience of COVID-19 indifference:

“*Some colleagues no longer wear their masks and gloves and they said that they are truly fed up with this situation. They say that they are self-censoring, and clearing their minds that there is no such a disease, when there really is.*” (Male nurse).

Another nurse commented:

“*I’ve been so obsessed with the issue of personal protection that I’m fed up, and now I’ve become indifferent.*” (Female nurse).

### Responsible self-protective reactions

According to analysis of data, responsible self-protection was another one of the nurses’ reactions to providing care for patients with COVID-19. This reaction was shown through moderation in providing care, self-supplying protective equipment, and observing standard precautions. By reducing duration of care and establishing limited contact with the patient, nurses tried to minimize exposure to the patient as much as possible to protect themselves. They argued that reducing the exposure time does not mean reduced quality of care, but high-speed care. One of the nurses explained their experience this way:

“*I try to do any care or procedure in the shortest possible time, but in a proper way, so that I can leave the patient quickly.”* (Male nurse).

Another nurse said:

“*It’s not a priority to communicate with the patient in these circumstances, and it can be moderated because transmission may also happen through talking. That is why we try to minimize contact with the patients, and do the essential tasks.*” (Male nurse).

Self-supplying of PPE meant purchasing PPE from the open market, which incurred a huge cost to nurses as hospitals provided limited number of PPE, and officials demanded personal protection requirements. One of the nurses said:

“*The hospital does not easily provide us with protective equipment. I had to fork out 700000 or 800000 Tomans to buy masks, gowns, caps, goggles, and special clothing out of my own pocket. Besides, it was a trying experience to find many of them in the open market.”* (Male nurse).

Another nurse explained:

“*We ask authorities and the nursing office for equipment every shift and every day. They give you more equipment if you peruse it, and if you don’t, you have to put up with what you are given in the ward. Our ward head nurse is very stingy in dishing out equipment, as if our lives are not important for her.*” (Female nurse).

By observing the standard precautions (personal safety and protection principles and taking care when providing care), the participants tried to assume responsibility for their own protection. One of the nurses described their experience as follows:

“*I wasn’t afraid or scared of providing care for patients with COVID-19, but I was careful; fear leads to death. I was very cautious in providing care for these patients and carefully observed all principles of personal protection.*” (Male nurse).

## Discussion

The themes emerging from analysis of data was an attempt to achieve the study main objective, namely searching, exploring the protective reactions of ICU nurses when providing care for patients with COVID-19. The themes extracted answer the question how nurses experience protective reactions when providing care for patients with COVID-19.

The unbalanced (irresponsible) self-protective reactions were one of the main reactions of ICU nurses. Selfish, obsessive, and indifferent reactions were the unbalanced reactions exhibited by them when providing care for patients with COVID-19. Providing care for patients with COVID-19 is a stressful experience for ICU nurses and incurs severe psychological pressure to them [16, 17], and living with stress means living to survive. Hence, this problem per se can provide an explanation for the nurses’ selfish self-protection.

Another one of the ICU nurses’ unbalanced reactions for self-protection was COVID-19 obsession. ICU nurses are at the frontline of the fight against COVID-19 and in the center of crisis. The nurses’ concern about being infected and transmitting this infection to their family members tremendously increased their anxiety and fear [18]. Excessive anxiety and fear with regard to infection with COVID-19 can exacerbate their obsessive thoughts, which may lead to compulsion in these nurses, so that they overdo the protective and hygienic practices; for instance, they could wash their hands and disinfect equipment for hours, leading to loss of energy and fatigue. This fatigue makes them refrain from observing hygiene issues and exposes them to the risk of developing COVID-19.

The most dangerous unbalanced reaction of ICU nurses (especially when providing care for patients with COVID-19) was COVID-19 indifference, which resulted from fatigue due to wearing PPE and observing the principles of personal protection. In such circumstances, the participants self-censored and wiped the disease off their mind. This reaction can entail highly dangerous consequences for the individual, the family, and the community in general. Therefore, it is imperative that medical authorities and nursing managers monitor such an unbalanced reaction in nurses, and adopt preventive strategies.

Another theme obtained from analysis of interviews with regard to the ICU nurses’ protective reactions when providing care for patients with COVID-19 was responsible self-protective reactions. Since nurses had found themselves responsible for their own protection and also providing quality care for the patients, they tried to achieve this by moderating their contacts with the patients, supplying their own protective equipment, and observing standard precautions.

By reducing the duration of care and moderating their contact with the patient, nurses tried to reduce their contact with the patients as much as possible. In their study, Hang et al. emphasized the nurses’ need to avoid unnecessary contact with the patients in order to minimize transmission of the infection [18].

The lack of PPE is a problem experienced due to the increasing demand of the healthcare team, improper distribution of resources, and the lack of crisis-oriented vision in similar epidemics [17, 19]. According to the participants’ experiences, faced with shortage of PPE, many nurses procured them in order to protect themselves and their family members. On the other hand, they compelled hospital authorities to procure and supply PPE through their frequent follow-up, even for a few pieces of PPE.

Another responsible self-protective reaction was observing standard precautions by ICU nurses providing care for patients with COVID-19. Standard precautions included such measures as washing hands, using PPE, performing safe injections, and respiratory precautions. Being careful and observing the principles of personal protection in providing care made nurses assume responsibility for patient care without any fears, and fulfil their professional duties [8, 20].

## Conclusion

The present study results showed that nurses exhibit different self-protective reactions when faced with an epidemic and lack of PPE. These reactions may be unbalanced or responsible. By recognizing these reactions, high ranking health officials can promote positive reactions among personnel and reduce unbalanced reactions by finding their root causes, and then manage reactions expected of nurses in similar crises, since mental and physical security of nurses directly affects the quantity and quality of care they provide.

### Limitations

The present study only explored the protective reactions of Iranian ICU nurses. Although given the nature of qualitative studies, the researcher is not concerned about generalizability of the results. Nonetheless, it is recommended that similar studies be conducted in other contexts. Also, this study was a brief study, and prolonged engagement with the participants can provide a worthwhile means for understanding the nurses’ protective reactions during the COVID-19 epidemic.

## Supplementary Information

**Additional file 1:.** Interview Guide.

## Data Availability

The datasets used and/or analyzed during the current study are available from the corresponding author on reasonable request.
