# Supplementary material for: Protective reactions of ICU nurses providing care for patients with COVID-19: a qualitative study
Source: BMC Nurs. 2021 Mar 17;20:45. doi: 10.1186/s12912-021-00567-6 (PMC7968575; doi:10.1186/s12912-021-00567-6)
Supplement: Supplementary file 1 — Additional file 1:. Interview Guide. [file 12912_2021_567_MOESM1_ESM.docx]

**Additional file 1.** Interview Guide.

In this qualitative study, semi-structured individual interviews were used to collect data. In this type of interview, the questions varied according to the participants' answers. However, here are some of the questions that guided the interview in the present study and below these questions, It is mentioned as follows:

"Talk about the care experience of patients with COVID-19".

"Talk about the reactions and protective measures you and your colleagues conducted while caring for patients with COVID-19"

"Does the way you provide care change when dealing with patients with COVID-19?" please explain.
